# Designing Biodiversity Systems via Digital Kinships: Insights from Community Data Processes and Creative Practice

**DOI:** 10.1007/s10606-025-09524-2

**Published:** 2025-06-16

**Authors:** Michelle Westerlaken

**Affiliations:** Massachusetts Institute of Technology, Cambridge, United States

**Keywords:** Biodiversity technology, Data practices, Digital twins, Simulations, Community engagement, Participatory design

## Abstract

This study details how digital biodiversity data is used and gains meaning in local restoration projects, how these experiences contrast with large-scale innovation patterns, and what new design recommendations emerge from these insights. Digital innovations in biodiversity technologies are increasingly complex, fast-paced, and driven by technological capacities where data generation rather than biodiversity restoration risks becoming the primary goal. Focusing on a biodiversity restoration project with a living lab community in the Netherlands, this participatory research critically examines how plans for emerging technologies, such as biodiversity simulations and digital twins, contrast with local user relations to biodiversity data. Building on qualitative insights from six-months of fieldwork, a digital and physical data portal was designed to simulate ongoing technoscientific innovation and make their complex effects experientially available to users. Findings are brought directly in conversation with emerging technical features through four distinct themes with the aim to share user-insights and produce design recommendations for: environmental storytelling, prediction and future making, interactive dynamics, and simulation aesthetics. These themes articulate the community's preferences towards digital environments that support their nuanced, complex relationships with local biodiversity, suggesting a shift from top-down technocentric approaches to more community-driven and restoration-focused models. Based on this study, design recommendations are articulated for each of these four themes contributing detailed empirical and practice-oriented insights that propose how new biodiversity technologies can resonate more effectively with local biodiversity restoration efforts.

**Supplementary Information:**

The online version contains supplementary material available at 10.1007/s10606-025-09524-2.

## Introduction: Emerging Biodiversity Technologies

Over the last decades, the digitalization of biodiversity knowledge has fundamentally changed global strategies for understanding and mitigating biodiversity challenges. These technologies aid the collection, archiving, and interpretation of ecosystem and species data, thereby enabling humans to work with vast databases to uncover patterns and produce new knowledge (Waterton et al. 2014; Besson et al. 2022). Examples of such biodiversity technologies include complementing manual species observations in the field with acoustic or camera sensors, digital archives that structure large amounts of biodiversity information, algorithms to calculate and predict ecosystem responses over time, satellite and sensor networks that provide data on environmental changes and species movements, and digital infrastructures such as cloud-based systems that make data accessible to a variety of users (Bakker and Ritts 2018; Gabrys et al. 2022). More recently, the availability of supercomputers and algorithms that can perform calculations on increasingly large amounts of data from different digital sources are contributing to the development of biodiversity simulations such as digital twins and virtual infrastructures that connect different types of sensor data and archives. (European Commission et al. 2020; Jiang et al. 2021; Barbie et al. 2022; De Koning et al. 2023). These digital simulation initiatives have the potential to transform understandings of biodiversity not merely through new or separate data streams but also by enabling people to enter into and engage with virtual environments that represent ecosystems from already computationally combined environmental datasets.

Biodiversity monitoring technologies are developing rapidly, as planetary ecological crises intensify and economic development opportunities for automation and offsetting infrastructures increase. Global assessment and restoration frameworks, technological innovation by startups, publicly funded monitoring projects, and regulations for corporations are also rapidly implemented. This fast-paced innovation landscape makes it challenging to monitor the effects of these technologies on our understanding of biodiversity and understand the new risks that they may pose. Prior research shows that new biodiversity technologies are often informed by new technological capacities – a predominantly top-down approach – rather than driven by user needs in ecological monitoring and restoration projects (Westerlaken 2024). This has serious consequences that determine what biodiversity data is being generated and which ecosystem elements are becoming more datafied. Those species who make sounds such as bats and birds are increasingly measured through acoustic technologies; or those species that can be seen on satellite data such as tree canopies become more and more recorded; but these innovations risk overlooking other important ecosystem dynamics (ibid.). Moreover, the sheer volume of data produced through monitoring technologies does not necessarily correspond to an increase in ecosystem restoration or prevention of biodiversity loss. To ensure that data collection does not become an end goal in itself but only a means towards actual biodiversity restoration, more research is needed to critically evaluate counterproductive types of innovation and bring emerging technologies more directly in conversation with ongoing biodiversity restoration projects.

Technologies such as digital twins and large-scale data infrastructures are complex systems, which makes it challenging to involve potential users and biodiversity restoration communities in their design processes. Discussing the inner workings and potential of these innovations first requires making their implications experientially available to the users and communities affected. This project thereby expands ongoing ecological research in CSCW by using relational participatory structures and creative practice to connect emerging technologies to local biodiversity restoration projects with the goal to inform the creation of more user-driven innovation in biodiversity restoration. Working with an ecovillage community in the Netherlands, this study articulates ethnographic insights from a six-month participatory research project. This research thereby asks how digital biodiversity data is used and gains meaning in local restoration projects, how these experiences contrast with large-scale innovation patterns, and what new design recommendations emerge from these insights.

## Related Work: Technoscientific Innovation Critiques and a Need for Alternative Proposals

The consequences and implications of digitalizing knowledge on biodiversity and ecosystem processes have been addressed in fields such as Science and Technology Studies, Media Studies, Human–Computer Interaction, and CSCW. Researchers have produced critical analyses of digitalized biodiversity and planetary science initiatives on a variety of issues for decades (Bowker 2000; Vertesi and Dourish 2011; Turnhout et al. 2013; Büscher 2016; Whitelaw and Smaill 2021). It is clear that an increased scientific understanding of biodiversity has produced valuable knowledge that can potentially aid in preventing biodiversity loss and restoring ecosystems. Yet, the measuring and monitoring of biodiversity has also been critiqued for being reductionist, fragmented, and overly embedded in economic trade (Bowker 2007; Ellis et al. 2010; Yusoff 2010; Büscher et al. 2012; Turnhout et al. 2013). These works illustrate how biodiversity conservation risks predominantly focusing on obtaining large datasets and building systems in which biodiversity rates are commodified and can be exchanged as human-centred ecosystem services. The idea of ecosystems as entities that provide services, while sometimes helpful in shaping nature protection rhetoric, reinforces an anthropocentric paradigm that seeks to measure complex ecosystem processes in terms of their fragmented instrumental values (McElwee 2017). Over the last few years, digital twin phenomena and other recent computational infrastructures have been critically analysed: these systems use considerable energy resources, risk reinforcing existing inequalities, are predominantly funded and developed in the Global North, and risk producing or confirming one-sided data (Nadim 2021; Gabrys et al. 2022; Nost and Goldstein 2022; Pritchard et al. 2022; Westerlaken 2024). Each of these texts advocate for more pluralistic, multidimensional, or relational technological systems that can better account for the complexity of the ecosystems they represent and the socio-political contexts in which they are used. Yet, despite these critiques, digital innovation patterns continue to remain predominantly driven by technoscientific advancement that prioritizes data generation as a goal in itself over actual biodiversity restoration.

Contributing more practical examples, researchers in the field of Design, Human–Computer Interaction, and CSCW are experimenting with producing and reflecting upon new digital systems and critical data practices that could incorporate alternative design qualities, thereby potentially inspiring new approaches to the development of environmental technologies (Fortun et al. 2016; Gabrys 2016a; Sheikh et al. 2021; Dolejšová et al. 2023; Longdon et al. 2024). Biodiversity data is an area in need of more experimental prototypes to reflect upon socio-technical dimensions of biodiversity preservation efforts that can not only critique counterproductive types of innovation but also generate alternative proposals. This project aligns with such a generative orientation and seeks to contribute community-driven design recommendations for biodiversity technologies. Recent research in CSCW on ecological transitions and cooperative design practices emphasize notions of ecological relationality and entanglement as both a methodological and ontological position in research projects to question techno-economic values (Light et al. 2024). This type of work connects to previous related research on situated practices in design (Suchman 1993) and relational studies of participatory infrastructuring (Star 2000; Bødker et al. 2017). However, environmental technologies and data infrastructures are currently being developed at global scales through automation and datafication patterns that make it difficult to engage them in participatory studies because their effects remain largely invisible to users. This project therefore adopts participatory research methods that simulate complex data infrastructures at a local scale to make them experientially available to users and gather insights to generate new design recommendations for biodiversity technologies. To do so, this work aligns with existing research that attend to data in use, including the ways in which socio-material frictions of situated data practices emerge (Bates et al. 2016; Loukissas 2016). Using new creative practices to attend to digital biodiversity data infrastructures, this study aims to produce more detailed empirical materials on ecological transitions that can challenge the fast-paced technoscientific innovation that currently determines the development of biodiversity monitoring. Critical analyses of emerging design patterns and empirical details of biodiversity data practices thereby must be addressed together to contribute towards generative design research that incorporates more effective, relational, and participatory approaches to biodiversity technologies.

This paper is methodologically grounded in practice-based design research where the use of data as a design material facilitates participatory processes through which future practices are imagined and discussed (Giaccardi 2019; Huron et al. 2022). The work furthermore aligns with and extends CSCW methods that investigate the plurality of data practices in relation to environmental data and (more-than-human) communities (Niederer and Priester 2016; Dema et al. 2017; Young and Lutters 2017; Light et al. 2024). The notion of ‘digital kinship’ emerged as an important analytical lens in this study to describe what the community articulated as the most effective or meaningful engagements with digital biodiversity data in community restoration projects. This concept is grounded in existing scholarship on ecological ‘kinship’ that re-imagines the relations between human and more-than-human entities in biodiversity conservation (Greeson 2019), citizen science projects (Dunkley 2023), or to inform new legislation (Strang 2023). This concept thereby serves to connect this paper’s four empirical sections and emphasize the shared quality between them with a focus on the relations that community members articulated between digital data and ecological phenomena. The findings are brought directly in conversation with emerging technologies to highlight the contrasts between technoscientific proposals for biodiversity simulations that are currently developed and more user-driven biodiversity concerns.

Local communities with specified biodiversity restoration plans and objectives are an important group of users of digital biodiversity technology. They undertake the practical work involved in caring for their local lands and they are expected to implement biodiversity policies. The effects of new computational technologies are becoming apparent on a local level within these spaces. In these local contexts, attending to data practices helps to identify which types of data are relevant and impactful and how users engage with biodiversity data in more detail. The goal of this study is to articulate design recommendations for biodiversity technologies and systems that are less technocentric in their development and more in line with the needs and preferences of their users, including local communities who aim to care for biodiversity in their neighbourhoods. These findings extend research in CSCW and beyond by contributing practice-based research formats for understanding complex socio-technical dimensions of environmental data and producing user-driven design knowledge on how computational data infrastructures can resonate more effectively with local biodiversity restoration projects.

## Method: Building a Digital Data Portal with a Local Community in the Netherlands

The fieldwork for this project was prompted by more extensive research into ongoing developments in biodiversity technology. Desk research and interviews with people working on biodiversity technology initiatives showed how large amount of public funding is invested in innovating digital technology in the global North, specifically in Europe, with the aim to address global biodiversity challenges (see e.g. AMMOD 2024; Arise 2024; BioDT 2022; Sovereign Nature Initiative 2024; ESA 2024). The Netherlands is notably invested in these projects, not only because of their focus on digital technology as a primary innovation sector, but also due to its particularly dire biodiversity situation. As a densely populated country and simultaneously the second largest exporter of agricultural products in the world, biodiversity has decreased excessively due to nitrogen emissions in the form of nitrous oxide and ammonia as well as other forms of ecosystem degradation (Berkhout 2018; Sanders et al. 2019). The European Court has now imposed strict limitations on the Netherlands, specifically on enforcing lower nitrogen emission levels (Stokstad 2019). This has caused a political and societal deadlock in the rural parts of the country, where urban and agricultural developments have now been largely halted, creating immediate knock-on effects on housing availability, economic welfare, and the rise of populist politics (van der Ploeg 2020). In this political climate, large scale investments in biodiversity technologies are portrayed as technoscientific solutions that will help to address environmental challenges (Westerlaken 2024).

**Figure 1.**
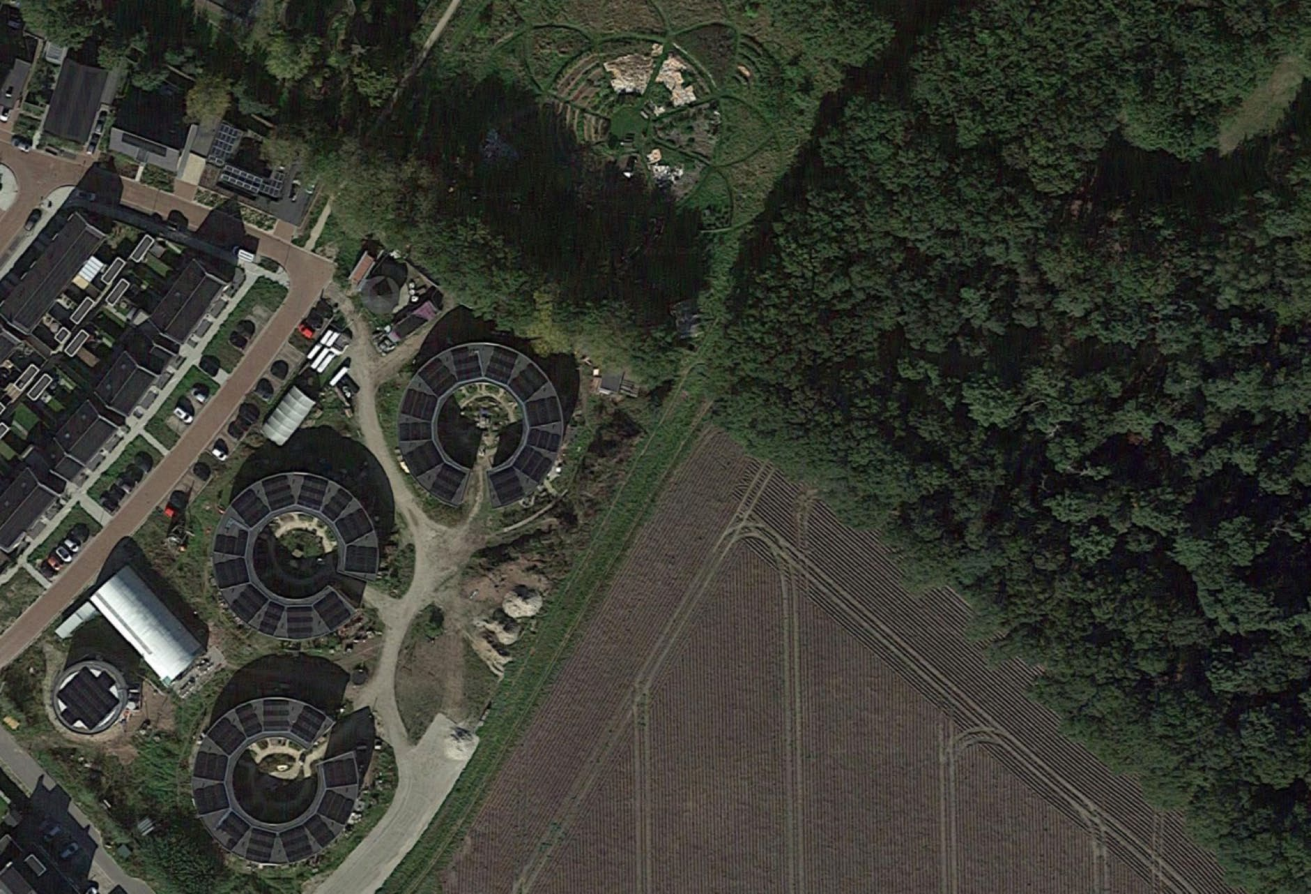
Satellite view of Ecodorp Boekel, an ecovillage with 62 inhabitants in the southeast of the Netherlands, situated at the border of a small town, in between farmlands and a small, protected forest.

In the midst of these tensions and large-scale technological investments, some rural communities are creating initiatives that can address societal issues at a local level. One such initiatives is ‘Ecodorp Boekel’, an ecovillage and living lab in the south-east of the Netherlands where people spent the last 12 years developing and reflecting upon sustainable forms of living (Ecodorp Boekel 2024). The 36 rental homes and food forest/garden are located on a two-hectare site and situated between farmland, a protected forest site, and the border of a small village (Figure 1). The 62 inhabitants of the ecovillage are aged between 0 and 78 years old and come from different socio-economic backgrounds, aided by the affordable rental prices of their homes. They often work part-time and are expected to invest voluntary labour into the communal development of the ecovillage. Cooperative work in this project thereby aligns with recent CSCW research into its ecological entanglements and consist of community practices that produce human and more-than-human relations through voluntary labour in times of ecological crisis (Light et al. 2024). As a living lab and one of the most technologically advanced ecovillages in Europe, this is a particularly interesting site to further address new biodiversity technologies.

Over the last decade, this site has become a testbed for a variety of sustainable innovations including bio-based housing materials that store CO2, an on-site battery that stores energy from 600 solar panels, underground wastewater recycling systems, and architectural designs that encourage social cohesion. By practically experimenting with more sustainable futures, this site has the potential to inform large-scale innovations in ways that are receptive to both new technological developments and local community perspectives. Inhabitants are predominantly Dutch, and the ecovillage also includes two homes for people with refugee status, as well as several homes for care-dependent people. Most residents have a specific interest in topics related to sustainability, are often knowledgeable in topics such as ecology, permaculture, herbal medicines, communal living, health, Indigenous knowledge, and biodiversity. Residents select most new inhabitants of the village themselves, and their communication and cohabitation practices are guided by continuously developing community-based methods. This summary shows how this community is at the same time more diverse than its rural surroundings but also risks becoming homogenous in terms of its values towards sustainable practices and community living. This community is also already well-versed in undertaking participatory activities and engaging in democratic decision-making, which enables design researchers to document a wide variety of community proposals as participants are used to contributing different ideas and perspectives.

In the spring and summer of 2023, I conducted extensive fieldwork at Ecodorp Boekel over a six-month period, including four in-person visits: one introductory daytime visit, followed by stays of 3.5 weeks, 2 weeks, and 2.5 weeks. Between these visits, I collected remote biodiversity data, undertook remote interviews, prepared fieldwork activities, and developed design materials through iterative processes. I maintained regular contact with community members through virtual one-hour meetings, email, and Signal messaging. The ecovillage newly created a 10-year plan for local biodiversity, and I was curious to learn how innovations in biodiversity technology impacted this community. Thus far, the ecovillage’s data practices consisted of an ecologist-led annual species count as well as informal qualitative observations on changes in local biodiversity. The community showed interest in broadening biodiversity monitoring through other technologies but mentioned that these methods are too costly or time-consuming to implement. During the first three months of this project, we collectively developed a more comprehensive understanding of the local biodiversity using a variety of technologies including camera traps, acoustic sensing methods, citizen science apps, as well as interviews with visiting experts, forest walks, and several community workshops. These activities were all organized together with the ecovillage inhabitants and their extended network. Alongside this fieldwork, I undertook interviews with people at different digital biodiversity initiatives on a national level to gather knowledge and local data through online platforms, maps, and other connected open data initiatives (also see Westerlaken 2024).

After this period of participatory data collection, I presented this biodiversity data back to the community through the creation of a digital/physical data portal, installed at the ecovillage. This installation, called *Digital Kins: A Biodiversity Data Portal*, is designed to expand technoscientific practices of automation and datafication by investigating how local biodiversity data is used and gains meaning within communities while building relations of multispecies kinship between humans, other species, sensors, and digital data. *Digital Kins* is both a wordplay on digital twins to identify alternative modes of engaging with biodiversity data as well as to reconceptualize biodiversity data to build kinship relations between humans and other living entities. Kinship, in this reading, aligns with both science-based as well as Indigenous conceptualizations of ecological relations that extend into multispecies dimensions to re-imagine communities by including the non-human inhabitants of ecosystems (Greeson 2019; Dunkley 2023; Strang 2023). The data from other living entities in this installation thereby became prompts for considering the needs and interests of non-human beings and living systems for collaborative future-making and biodiversity restoration activities in the ecovillage.

**Figure 2.**
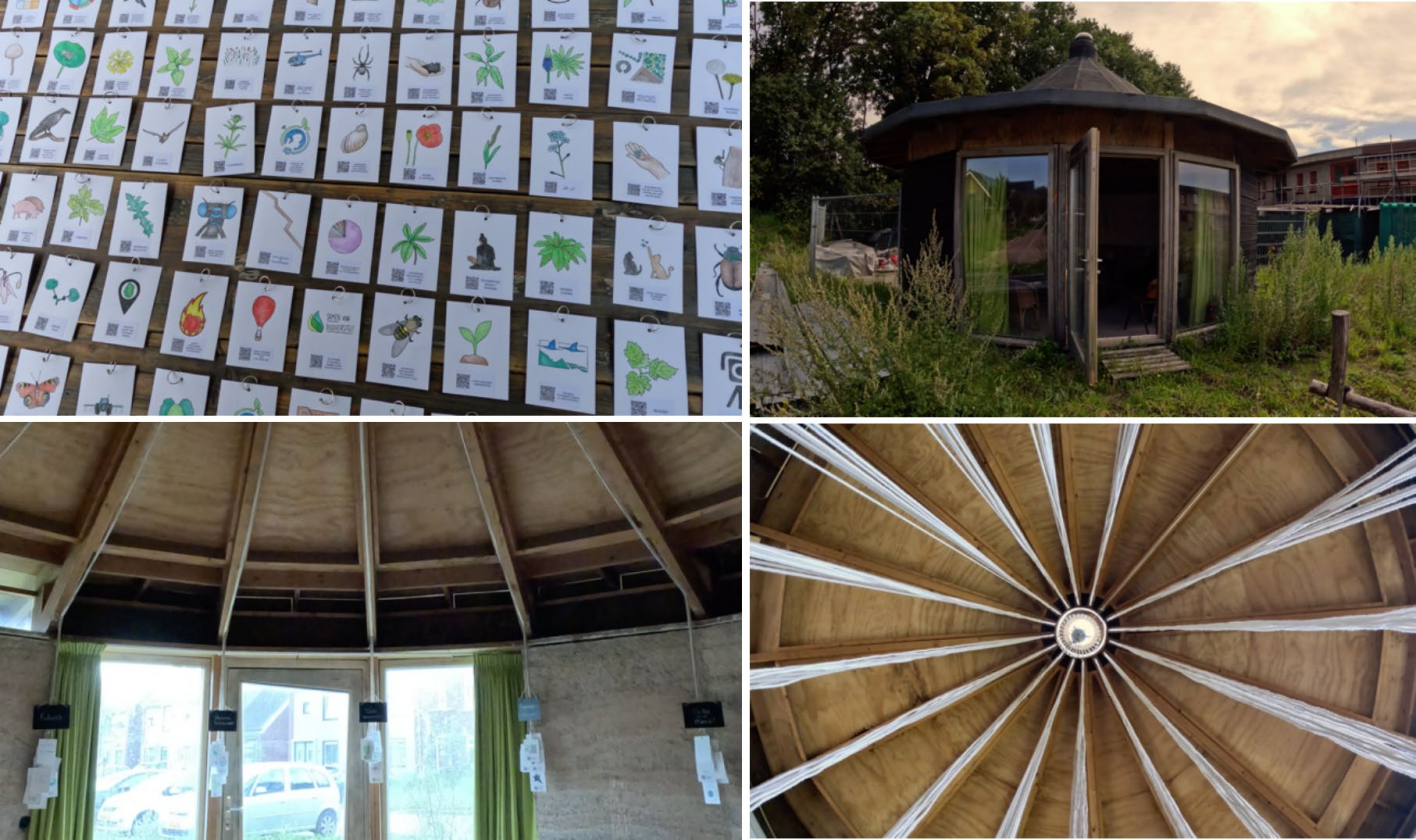
Pictures of *Digital Kins: A Digital Data Portal*, installed at the ecovillage. Each of the data points can be rearranged and hang from the circular roof structure of the building through 4 m long cotton ropes. The supplementary video file shows this installation in more detail.

The installation itself consists of 101 data points – in the form of hand-drawn illustrations with QR codes – that each connect to their online open-data sources (Figure 2, also see the supplementary video). These sources include data such as species observations, sound recordings, geospatial data, camera trap footage, recorded conversations, biodiversity platforms, reports, and stories shared by people in the local community. Environmental, species, and geospatial data were linked to the open-access infrastructures through which they were recorded. Other data was primarily stored on an open-source data platform that was specifically developed for this research project (see Westerlaken et al. 2022). Each data point connects with a cotton wire to the circular roof structure of *'Het Expo Huisje'* at Ecodorp Boekel. The wooden structure of this building, with its 5 m high conical ceiling, formed the inspiration for new modes of exploring, categorizing, discussing, expanding, and questioning the meaning and usefulness of data practices with local participants at this site. Besides all the conversations that emerged while gathering data and building the data portal, seven structured community workshops with a total of 27 participants (3–5 people per 1,5 h session) were hosted to encourage exploration, to critically discuss data practices, and to add new findings to the installation through an additional 59 data points. These workshops were divided in three main parts: (1) a discovery phase where participants used their phones to access the QR-code data points and share findings with each other, (2) a sense-making phase where participants created their own methods for grouping and cataloguing data based on their interpretations, and (3) an expanding phase where participants enriched the installation by adding new data, expanding the biodiversity archive. Each of these parts ended with reflections shared by participants. All seven workshops were audio-recorded and transcribed.

## Data Analysis: Existing Design Patterns in Conversation with User Experiences

The project’s research data – besides all the biodiversity data that was included in the installation itself – consists of interview and workshop transcripts from audio recordings, daily ethnographic journal entries from all fieldwork visits, workshop notes, and audiovisual materials documenting both fieldwork and workshop activities. The subsequent analysis of this data was also informed by prior desk research into the current innovation landscape of new biodiversity monitoring technologies as well as interviews with professionals working in this sector (see also Westerlaken 2024). This prior research identified key biodiversity innovation patterns such as the increasing anticipation of predictive ecosystem algorithms, new species identification tools, digital response mechanisms that can intervene in ecosystems, and the technoscientific quest for increasingly high-resolution sensors and satellites. Interview and workshop transcripts were analysed using *Atlas.ti* (for remote interviews) and *Trint* (for in-person recordings) to identify conversation segments that challenge these mainstream innovation approaches and highlight contrasting dynamics in the communities’ interactions with biodiversity data. The analysis of this project’s data thereby focused on bringing local perspectives in conversation with dominant technoscientific design patterns and available scholarship on these topics.

The following sections of this paper each narrate findings from this project across four thematic areas that combine observations on biodiversity innovation patterns with community discussions and activities. These four themes include (1) environmental storytelling, (2) prediction and future-making, (3) interactive dynamics, and (4) simulation aesthetics. Theme 1 and theme 4 began to surface early, already visible in handwritten fieldnotes. Tensions between emerging biodiversity technologies and community data practices stood out clearly in relation to these two themes and were raised by participants throughout different fieldwork activities. For example, the question of how local concerns were reflected in biodiversity data strongly shaped data storytelling discussions in several workshops. Secondly, aesthetic contrasts between biodiversity technologies and lived experiences also stood out from the very first day of fieldwork in March, marked by torrential rain and local flooding, to the final fieldwork day in August, when a heatwave and drought brought serious ecological and health risks to the community. On the other hand, theme 2 and 3 emerged later in the data analysis process, when empirical data was qualitatively coded in relation to emerging design patterns and it became apparent how issues around ‘prediction’ and ‘interactivity’ thematically aligned.

This process led to important practice-oriented findings that ask how digital biodiversity data is used and gains meaning in local restoration projects and how these experiences contrast with large-scale innovation patterns. These findings do not only stem from the community data in this project but also draw connections to existing scholarship in fields including Science and Technology Studies, Human–Computer Interaction, CSCW, and virtual worlds research with the goal to produce new design recommendations for engaging with biodiversity data through computational systems.

## Environmental Storytelling: The Creation of Meaningful Digital Experiences

Increasingly detailed digital simulations of environments and ecosystems can pull data from near inexhaustible sources. For example, such digital environments can incorporate camera trap networks and satellites that are recording high resolution imagery 24/7, entire DNA sequences of numerous species, detailed 3D models of ecosystem components, and large historical archives with other environmental data. A closer look at these systems shows that rather than presenting all this data at once, virtual representations of ecosystems are specifically designed to create manageable and meaningful experiences for different users. These systems are curated to enable users to access the data they are looking for. Such platforms thereby engage in a form of environmental storytelling where ecosystems become visible at particular scales and users can access different layers of information so that environments appear coherent and accessible for human understanding.

In research on virtual worlds, the notion of environmental storytelling is used to describe a narrative technique of conveying information and evoking response through the design and structure of the virtual environment, where different stories are made legible (Jenkins 2004). Environmental simulations inevitably contain simplified and mediated experiences of certain aspects of ecosystems, tailored to human experience via digital environmental storytelling techniques. As all technologies are designed by humans, these simulations primarily convey how humans perceive, monitor, and understand environments. Thus, the process of translating ecosystem data to digital representations for human users requires a form of anthropocentrism that is fundamentally at odds with the much more complicated and less coherent nature of the ecosystems it proposes to represent. Rather than trying to combat or obscure this inherent limitation, one that is structurally present in all forms of mediation, the more appropriate question that helps to question how emerging biodiversity infrastructures are designed is: what forms of digital environmental storytelling can shape biodiversity simulations in ways that are meaningful for the communities who will work with them?

**Figure 3.**
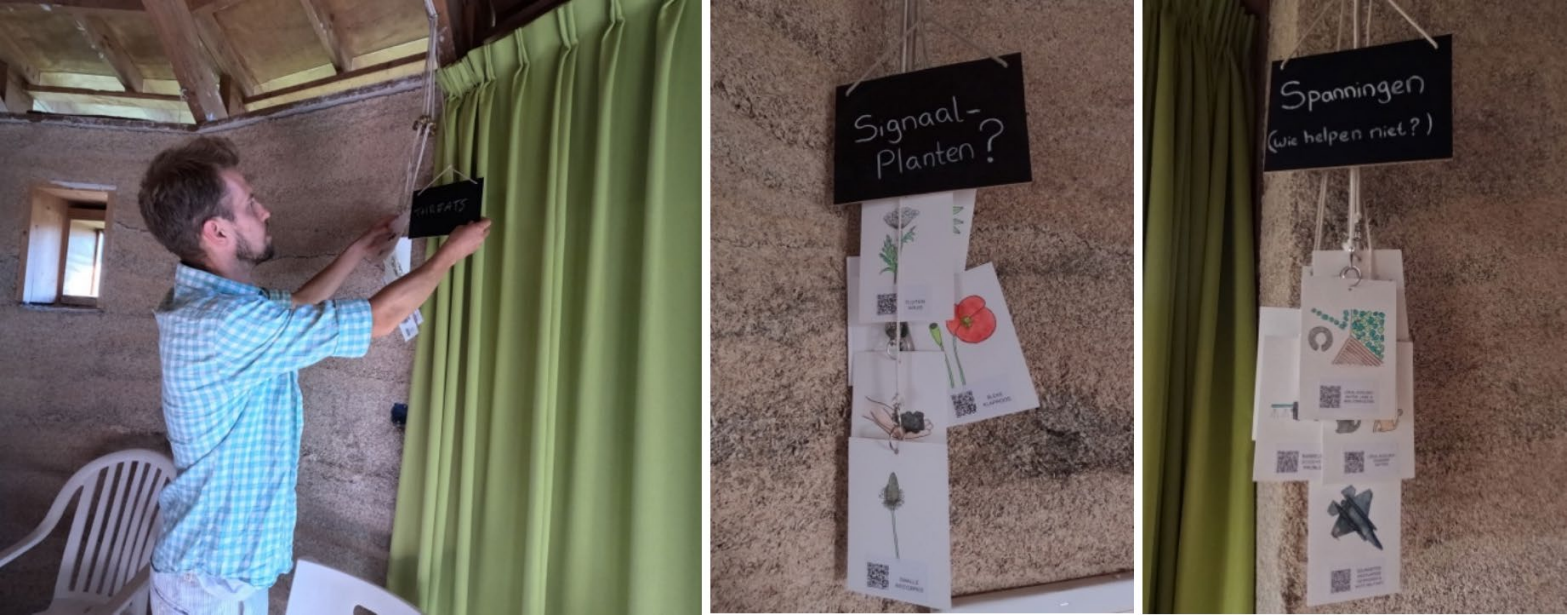
Participants grouped together different data points in categories named ‘threats’, ‘indicator species’ (signaalplanten), and ‘tensions’ (spanningen), while discussing the meaning and importance of this data.

Participants who engaged with the *Digital Kins* portal collectively reflected on the need to maintain the bigger picture of what is at stake *locally* to understand and improve biodiversity. While exploring the different data points, participants framed most discussions around the local environment they care for and discussed tensions regarding land-use conflicts, financial challenges, pollution, or other external factors that presently influence biodiversity at this site. When they did zoom in on a certain species that has been identified at the ecovillage, the data that mattered most showed what this observation could mean for future biodiversity *here*. E.g. “I don’t find any information about the red campion [a wildflower], only that it has been identified here. But what we want to know is whether it’s an indicator, or something about the type of soil [it grows in].” Instead, the data that was most commonly presented to the participants through the ObsIdentify citizen science app through which species observations were recorded contained general textual and geographical descriptions of these species and records of sightings in other places (ObsIdentify 2024). This data was largely ignored, and participants collectively discussed the implications of their observations in relation to the local land and community. To add more useful data to the installation, participants searched for complementary information through online search engines and added data to the portal about different types of indicator species to better understand the relation of *this* species to environmental factors such as the local soil quality or pollution levels. “The presence of one indicator species is actually not a sign of biodiversity in itself.”, one participant remarked*.* While single observations and general descriptions provide information, participants were seeking out less generalized formats that represented biodiversity data in ways they could trace back to their local environments, emphasizing the significance of tailored and relevant narration of biodiversity data (Figure 3). In other words, kinship relations with digital biodiversity data emerge when technology users draw more applicable connections to their species observations.

A second dimension on narrating digital environments that stood out from participants’ discussions focuses on using data to attend to local challenges. For example, one participant critically remarked how the use of camera traps may help to identify local bird life, but this data does not really help the ecovillage in improving its biodiversity as long as the neighbour farmer continues to use toxic pesticides that are damaging the air and soil quality of the ecovillage. They remarked that such factors are not visible on the camera trap footage. Local pollution levels and camera trap footage of birds may not appear to be connected to each other when looking at the data, but for this participant emphasizing these relations or the lack thereof is particularly important. They shared an experience of cycling past the farm and ‘being shocked’ by the overwhelming smell of pesticides. When encountering a data point in the installation in relation to farming pesticide use, they remarked that “oddly enough this data was not at all about that, which seems a bit of a mismatch”. On the one hand, they noted how a combination of different sensor sources into one data portal could provide more insights and correlations between local bird life and air pollution. However, this participant was mainly concerned that selecting partial data from this installation could paint an all too optimistic story of the local biodiversity while the tensions with local farming practices and other issues risk disappearing into the background. “Over here we’re trying to improve the biodiversity, but right next door they are destroying everything”. They further implied that instead of using camera traps, the ecovillage should focus on producing data that can better narrate the tensions between their biodiversity plan and the neighbour’s farming practices. Meaningful digital environmental storytelling, for this participant, emerges from including issues that are locally relevant. Merely browsing through a list of identified species or camera trap footage – which seems to be the proposal of many available biodiversity monitoring technologies – is not enough for users who experience the impacts of biodiversity loss. Here, digital kinship dynamics only emerge when users experience biodiversity data that narrate environmental concerns that are deemed relevant to local challenges.

The interactions of this ecovillage community with their biodiversity data show that producing more data does not necessarily lead to better identifying overarching patterns or ending up with more coherence. Participants themselves also selectively compiled and categorized available data to align with their environmental concerns and at the same time they worry that this enables people to construct more selective narratives. Despite the community’s homogenous ideas regarding sustainability, each of the workshops was characterised through vastly different conversations while engaging with the same data. These findings show the impacts that interactions with different forms of data can have on local communities.

Existing scholarship argues that technologies such as satellites and sensors capture the world in specific ways, thereby apprehending the environment narratively and making certain stories possible (Nadim 2016). Informed by Latour’s notion of the ‘circulating reference’, related work demonstrates how biodiversity data travels through digital infrastructures where metadata reproduces entities into different forms such as diagrams, documents, databases, and genetic barcodes, potentially making such transformations increasingly less visible (Latour 1999; Nadim 2021).

Digital technologies that simulate or mirror ecosystems are not neutral entities that share objective insights. Instead, this project reveals how they produce spaces in which selective storytelling inevitably emerges. Rather than a passive source of information, the data points in this project actively emphasized existing tensions and created new ones. These do not only include tensions around pollution, pesticide use, and what local information can be drawn from indicator species as described above but also emerged for example in discussions on local policy and potentially missing data that are illustrated in the following sections of this text. This local community preferred forms of digital environmental storytelling that clarifies the local implications of data and prioritizes data on the external factors that directly impact local biodiversity. Such insights further informs digital technologies to better respond to these demands by making digital narrative processes more visible to its users. Thus, rather than claiming to ‘twin’, ‘simulate’, or ‘mirror’ environmental processes, these infrastructures are better understood and designed more deliberately as tools for environmental storytelling through data created by sensors, archives, algorithms, ecosystem entities, and humans.

## Prediction and Future-Making: Data as Prompts for Discussions

What if biodiversity technologies can help to predict environmental changes? Longstanding environmental simulations such as weather forecasts already include such predictive qualities, but the possibility of predicting biodiversity potentially helps to guide policy and decision making towards future biodiversity restoration. The main advantage of virtual worlds and simulations that is advertised in this context is that ecosystem changes or human interventions can be experimentally modelled first in digital environments to try to foresee their impacts before they take place in actual ecosystems. Such digital environments enable humans to rehearse potential futures and derive knowledge from speculative scenarios.

On a local level, communities also engage in speculation and prediction by talking through different future scenarios based on available data. The installation and workshop format in this project gave participants a dedicated time and space in which new ideas could be proposed and discussed in relation to the local biodiversity data points:

It was August and mosquitoes were well represented during the workshops. The topic of the ‘ecovillage mosquito policy’ was brought up in various conversations, aided by data points about insect-tensions, annoying or dangerous species, and conversations about which species humans should help and which species should be prevented from multiplying in the ecovillage. “Did you know that in Spain people are not allowed to leave stagnant water outside their homes, to prevent mosquitoes?”, one participant remarked, insinuating that this could be something to consider for the ecovillage as well. A discussion followed on how such measures are rather impractical and how the changing climate in the Netherlands will likely increase the population of potentially more dangerous mosquito types carrying infectious diseases. Another participant created a new data category called “threats” and added mosquitoes together with other unwelcome species such as wasps and snails to this group. One person remarked how mosquitoes are also important to the ecovillage because they attract bats, and bats are important species that are specifically mentioned in the ecovillage’s biodiversity plan. A big question mark was added to the “threats” category to convey less certainty about what these threats are and for whom they pose risks. Another participant created a new addition to the installation by writing down “each threat is a new opportunity”. The data, the workshop format and discussions, and the actual presence of mosquitoes prompted participants to talk through different approaches and understandings of how to deal with mosquitoes while sidestepping immediate decision-making. 

The challenges of relying on prediction models for discussing environmental futures have been articulated by describing the risks entailed in creating scenarios that may hide uncertainties and inequalities in predictive practices (Albert et al. 2020; Tzachor et al. 2022; Lazaro and Rizzi 2023). Nonetheless, automated biodiversity monitoring and data analysis are often regarded as trustworthy sources for environmental governance (Thayyil 2018). These digital developments are of major importance to predicting the consequences of environmental changes and extreme weather events, and predictive capacities will play an increasingly decisive role in biodiversity and conservation policymaking.

Within this community, such speculative conversations are important for explicitly creating less-hierarchical governance structures and making sure that people’s careful and informed ideas and experiences are all considered (Copeland et al. 2023). But how can computational technologies aide this process without overruling important democratic discussions? In this example, conversations moved from mosquito sightings and policy towards wider conversations on dealing with changing ecosystems, invasive species, and environmental futures. By using the data points as prompts for discussion, community members showed that the key to creating such cooperative spaces with digital technologies is to not present environmental data as end points but as conversation starters. Meaningful prompts, or meaningful data, for informed future-making must therefore raise further curiosity and offer different ways of thinking through environmental challenges. Creators and researchers of virtual worlds and other immersive media have long been aware of the paradox in which exploration and curiosity is not prompted by large open-ended digital spaces and unbounded stimuli or data, but instead requires the inclusion of limited options, curious objects, and curated interruptions that draw attention (Murray 1998; Bogost 2016; Gómez-Maureira et al. 2022). Biodiversity simulations could better incorporate designs that can prompt further discussions, for example by including deliberate cues regarding the limitations of data, by enabling data curation as provocations for collective discussions, or by combining data with specific notes about ongoing political tensions, conflicted scenarios, or speculative questions that connect biodiversity data with wider societal challenges.

**Figure 4.**
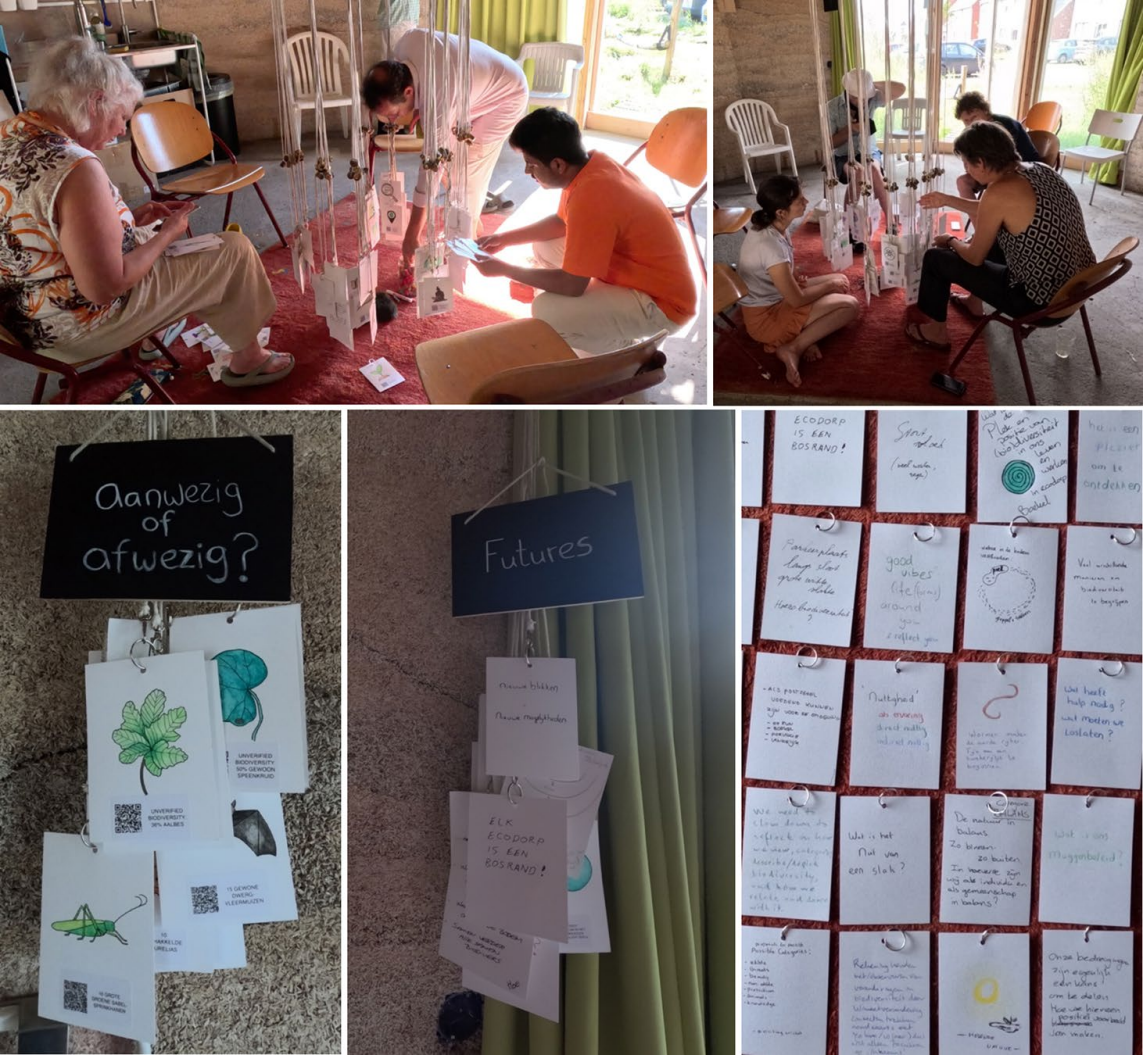
Images of the community’s engagement with the installation where participants used their mobile phone to access open data through the QR codes. Images of different categories created by participants: ‘present or absent?’ (aanwezig of afwezig), ‘futures’. Some of the notes and data points that were added by participants.

“If we don’t do anything for 100 years, there will be a beech forest”, one particularly ecologically knowledgeable ecovillage inhabitant remarked. “The question is, do we want that?”, she added. This response was prompted by a conversation around the question of “what if we do nothing and welcome all species?”. Such ‘what if’ questions around human environmental interventions, withdrawal, and rewilding are a frequent topic in the ecovillage, and different inhabitants have different ideas about how this should be done. Instead of making decisive future predictions, the data became part of speculative local policy discussions and made it clearer how residents think differently about managing collective spaces in the ecovillage (Figure 4). Digital simulations with predictive qualities do not just convey algorithmic calculations based on data repositories, but how this data is used has large scale impacts on the communities’ cooperative abilities to formulate diverse perspectives.

Rather than using predictive features to close down futures, more generative kinship relations with digital biodiversity data emerge when such data inspires further action and collective decision-making towards the most appropriate ecosystem interventions based on a variety of expert sources. In such contexts, digital technologies must be designed to enable asking ‘what if’ questions where data informs further discussions, learning, and relation-building rather than propose simple solutions. Digital environments could be shaped around this much more pluralistic practice of collaborative future-making by prompting further conversations rather than presenting predictive scenarios as the only possible outcomes.

## Interactive Dynamics: Multispecies Forms of Interactivity

What if digital biodiversity systems could use automation technology to directly intervene in ecosystems or autonomously respond to changes in the environment (Barricelli et al. 2019; Barbie et al. 2022)? A digital representation of the environment, in this understanding, does not just mirror certain aspects of ecosystems but can also respond within the ecosystems that are simulated. A digital simulation of weather changes that measures rainfall and flooding, for instance, can involve bidirectional activities between the ‘real world’ and the ‘virtual world’ by sending out early warning signals on extreme weather events (Riaz et al. 2023). Environmental digital technologies can not only passively collect data to represent ecosystems, but they also enable direct interactions between technologies and environments.

The issue that arises through this framing of interaction between technologies and ecosystems is that it limits the understanding of interactivity to a set of triggers or mechanical responses. When a sensor or algorithm detects a preconfigured event, a programmed digital or mechanical system is activated and triggers a subsequent response. This involves a rather narrow understanding of interactivity.

While engaging with the biodiversity data portal, one participant rubbed her arms and asked if “getting goose bumps from interacting with this data is also considered biodiversity”. Goose bumps, translated from the Dutch ‘kippenvel’ (literally: ‘chicken skin’), for this participant emerged as a bodily response to becoming more aware of her local surroundings through this data. The conversations in this particular group during the workshop developed into shared thoughts on how people engage with biodiversity as organisms themselves. Participants brought up how this installation could help to give biodiversity a place and meaning in their own daily lives through its kinship connections in everyday encounters with other species. “You are what you create”, one participant added. In this example, digital data generated environmental response in the form of goosebumps, leading to community conversations about the meaning of biodiversity in their daily lives, demonstrating a far wider conceptualization of interactivity between ecosystems and digital technologies. 

Available scholarship on the concept of ‘interaction’ reveals the much wider variety of social phenomena and agencies that can be included in understanding interactive systems (Rod and Kera 2010; Schleidgen et al. 2023). Various approaches grounded in Science and Technology Studies, New Materialism, and CSCW have furthermore described how relations between technologies and ecosystems involve much wider forms of interaction and entanglement where both digital and physical processes continuously co-constitute each other (e.g. Barad 2007; Gabrys 2016b; Light et al. 2024). These types of interactive relations also emerged through the local community’s engagements with biodiversity technologies and environmental entities and can help to broaden our understanding of a digital system’s interactive elements.

**Figure 5.**
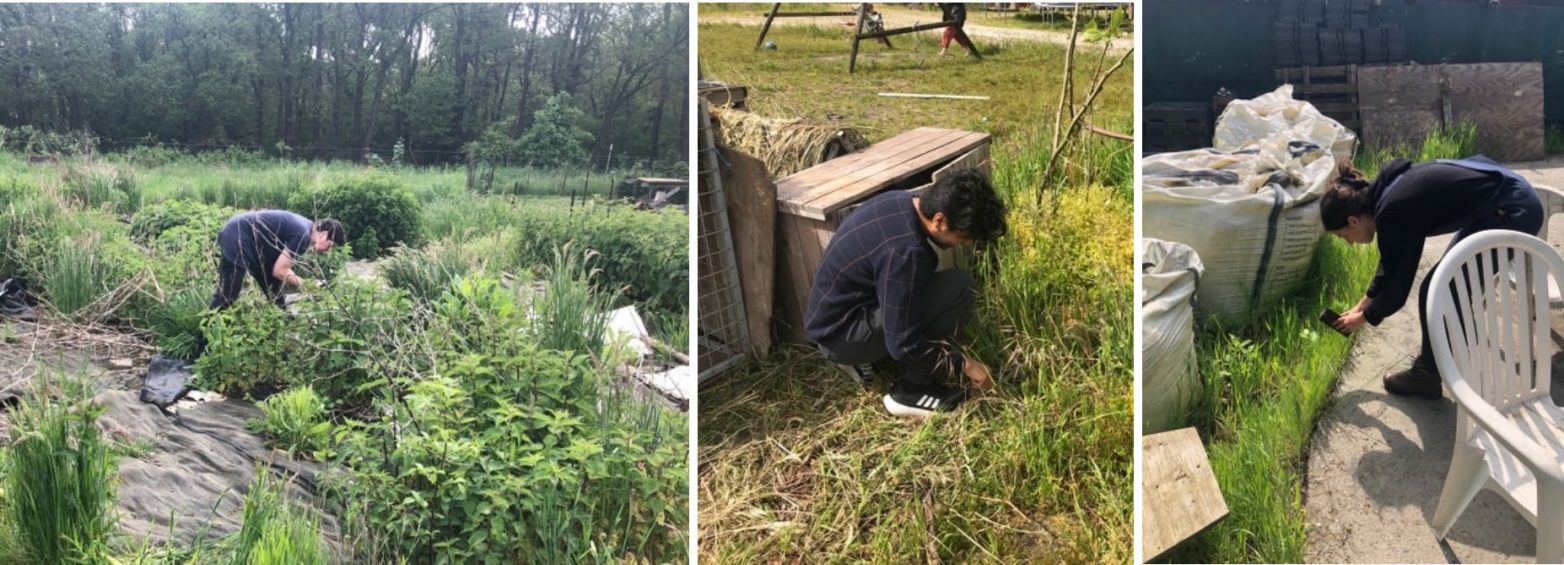
Images of the data gathering phase where community members use citizen science technologies such as *ObsIdentify*, which shaped different gardening practices.

Already during the data collection process earlier on in this project, participants became aware that biodiversity technologies were not a separate layer that simply documents the environment, but that they were themselves changing their attention and response to the species they observed through citizen science activities. One participant noted how using a mobile application to document and identify plant and animal species with automated image recognition was reshaping her gardening practices. She shared how finding and identifying new species through this app became a fun and addictive game which changed her movement patterns through the food garden. It also changed the way she observed species, now with the goal to picture them in ways that the image recognition algorithm would ‘favour’. She discovered how particular close ups and angles for plant species were more likely to receive a higher ‘rating’ in terms of the percentage of certainty with which the application identified photographed species. These activities spurred collective reflection on how automated species identification technology directly shapes interactions with the garden and with local biodiversity. It changed ways of moving through the garden guided by documenting new species, and it drew more attention to those species that looked like the image recognition algorithms would be able to detect. Grass-like plants and fast-moving insects were much harder to photograph and thus received less attention. This example shows how the combination of human use patterns and technological capacities to observe and document biodiversity directly shape interactivity with ecosystems (Figure 5). It demonstrates how digital technologies are already interacting with ecosystems and shaping human engagements, for instance through gardening practices with mobile apps. When developers of biodiversity technologies work to expand interactive responses, such as by triggering digital or mechanical interventions between technologies and ecosystems, it is important to understand these much wider interactive dynamics. When these are overlooked, digital systems risk becoming far too narrowly framed with regards to the effects and impacts they have on humans and ecosystems.

At several points during the community workshops with the data portal, different species directly participated in our shared interactions. Besides the presence of mosquitoes, other entities such as crickets, bugs, flies, wasps, pollen, traces of mould, cats, and dogs entered the data portal room. When they did, they often influenced conversations and attention towards the digital data, for instance by disrupting ongoing activities or becoming the topic of concern in relation to the data points. “Bats are too difficult to photograph”, one participant remarked. “But I see bats nearly every evening at dusk”, another participant responded, and they added a vivid description of the sounds their wings make while they hunt at night. Participants shared many stories of their encounters with different species in their everyday lives. Recognizing how other species are shaping data practices is fundamental to broadening anthropocentric conceptions of dynamic response between technologies and ecosystems to include multispecies forms of interactivity. 

For example, living entities in ecosystems, including humans and other beings, respond to and actively shape relations with technologies. Furthermore, technologies themselves shape relations with ecosystems. When new technologies are proposed with the goal to dynamically interact with ecosystems, interactivity must be understood in this much broader context. The ways that other entities participate in these processes disrupt human- or technology-centred understandings of interactivity and activates the potential for far more diverse ideas regarding the use of digital technologies to interact with environmental challenges (Westerlaken et al. 2023). With regards to biodiversity simulations, less technology-centred understandings of interactivity and response can guide the design of technology that better accounts for these diverse interactive modalities: how can goose bumps be integrated as feedback in digital systems? How can the different ways in which technologies shape human-ecosystem interactions become more visible? How can digital systems more deliberately recognize and include the dynamic responses of other species? These are just some of the questions that can shape new technologies, inspired by the attempts at forming digital kinship relations through the data gathering and gardening processes in this local community.

## Simulation Aesthetics: Kinship Relations and Complex Experiences

Besides the shocking smell of pesticides, the itchiness of mosquito bites, or an emotional response in the form of goosebumps, the creation and interaction with digital biodiversity data and technologies involves many other aesthetic components. Even though human experiences with biodiversity are deeply intertwined with elements such as beauty, taste, poetics, cultural relations, symbolism, meaning-making, physical encounters with other species, and various other aesthetic dimensions, there is often a disregard for these aspects in the creation of biodiversity technologies. This disregard leads to missed opportunities in creating more meaningful types of biodiversity innovation, because these aesthetics can inform much more relevant digital expressions of human-ecosystem relations.

The technoscientific focus of digital biodiversity solutions currently encourages a prioritization of scientific accuracy, efficiency, and quantitative output over design for other important aesthetic experiences. Interviews with technologists, scientific experts, and project managers who develop these technologies – nearly exclusively located in the Global North – show that these technologies are built with a predominant focus on their backend: developing data management infrastructures and computational capacities while the user experience and interface design are outsourced to hackathons, internships, or short consultancy projects (Westerlaken 2024). When their aesthetic dimensions do emerge through project presentations or prototypes, they are often characterized through visual designs that aim to convey futuristic digital worlds rich with vivid neon-like colours against dark backgrounds, sleek holographic elements, minimalist functionality and form, and high-resolution wildlife photography. Emerging digital technologies appear to follow a trend of creating highly detailed environmental realism which also increases the computational power and energy resources needed to run these simulations (Chang 2019). Notably, with the help of generative AI software that reinforces such aesthetics, these types of environmental designs are now becoming increasingly less cost-intensive to produce. However, these types of aesthetics starkly differ from the ways that local communities experience and engage with biodiversity. This became more visible through their interactions with the data portal in this project.

**Figure 6.**
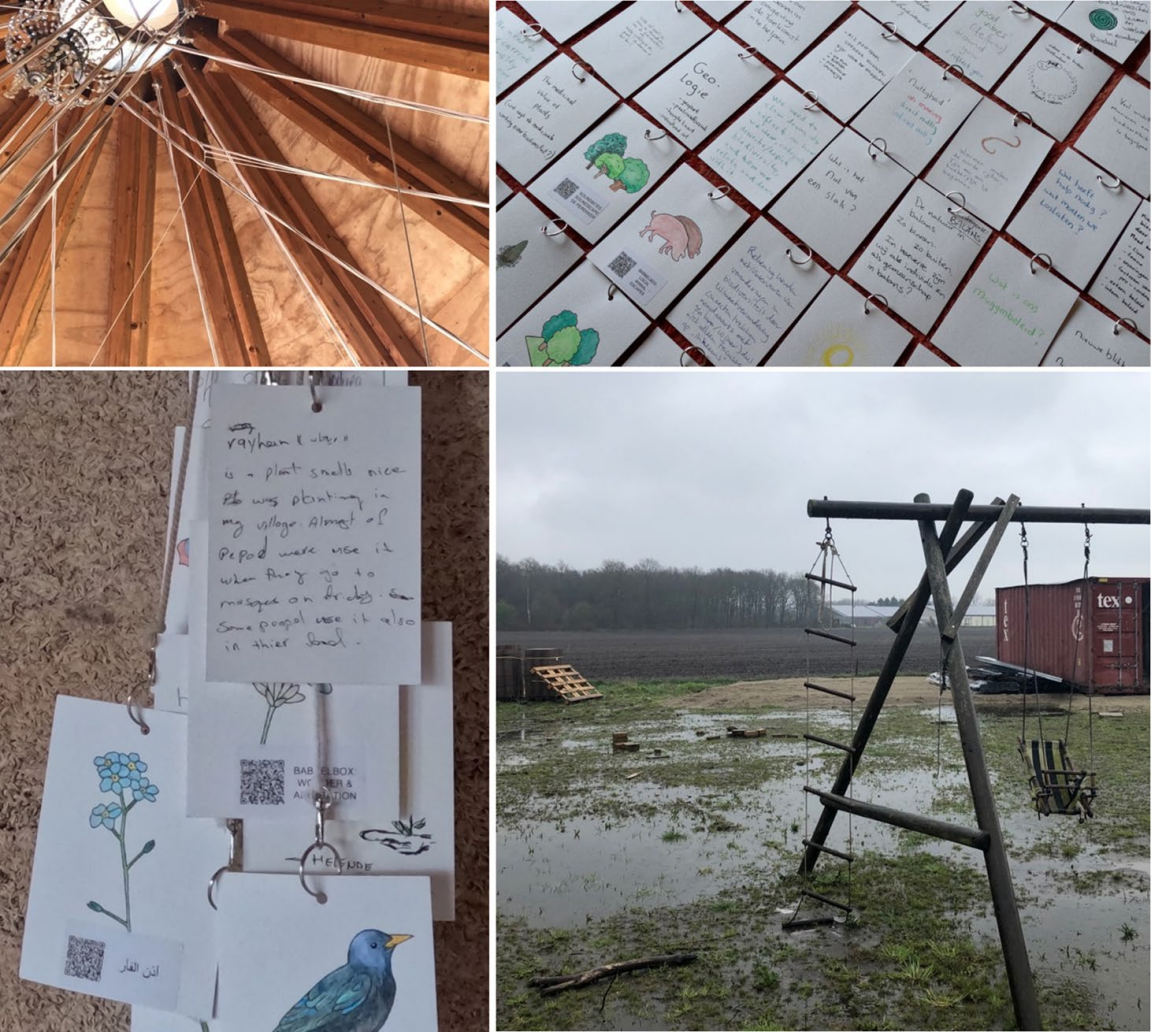
Images that reflect the aesthetics of the installation, including the knots created through restructuring different data points, some of the additional data points added by participants such as the inclusions of biodiversity knowledge from Yemen, and an example of the every-day, less stylized, life in the ecovillage-under-construction.

In designing this installation, I learned it was important to create an aesthetic experience that reflects the attention and care this community has for the local biodiversity. For example, during an earlier workshop about embodied biodiversity sensing in the data collection phase, community members shared the things they noticed about biodiversity, and this included descriptions such as vulnerability, strength, relaxation, freshness, richness, foundation, wind, the every-day, collaboration, shimmering, and movement. People reflected on the need to slow down to notice their surroundings and the physicality of biodiversity that goes far beyond the ability to identify different species to better understand biodiversity. One participant wrote how they noticed “a cocoon on a little thread, swaying in the wind”. These insights concretely influenced the design of the *Digital Kins* installation through a focus on spatially moveable data points, hanging from the tall ceiling in the middle of the space. These physical data points thereby became vulnerable to wind, humidity, and other local elements. The installation is built from recycled materials, where each data point (QR-code) is attached to a carefully detailed handcrafted illustration, thus connecting digital biodiversity data to design decisions that reflects this community’s relations to local ecosystems. Rather than futuristic minimalism or environmental realism, these were the aesthetic dimensions of biodiversity that were visible and expressed by this local community, and together with the architectural features of the ecovillage, they formed the inspiration for the design of the *Digital Kins* data portal (Figure 6).

When participants interacted with the data portal, they did not relate to any of the technology-centred aesthetic dimensions. Instead, they discussed how rewarding it feels to discover local biodiversity together, how they admired certain data, and they created their own categories to group biodiversity data called ‘beauty’, ‘love’, and ‘plants to cherish’. Participants were building their own community and kinship connections to the data. “There are a lot of plants here in the ecovillage. I don’t know a lot about it. But if I can, I try to remember if I’ve seen this in my country.” This participant, a young refugee from Yemen, then added a vivid description of an aromatic plant species (Rayhan, or ريحان(, which grows in the mountains of the lands he grew up in. This description includes details on the cultural use of this plant in traditional rituals and as food. He shared how he misses this particular plant for its smells and family memories. Even though this plant does not grow at this local site, it was important to him that these kinship histories with other species and lands were acknowledged by the community and reflected in the data. Through the plants themselves, the data points further evoked strong connections between our different pasts, presents, and locations. Important factors that are entirely ignored in most biodiversity technologies. 

Participants shared how some of the data made them feel deeply sad, such as pollution data or sound fragments of military planes flying over (this ecovillage is located close to a military airbase). In some cases, people got noticeably upset when they felt that the data may not be accurate or representative. Other participants got annoyed by reading other people’s notes that were added to the data portal, because they felt that their comments did not account for larger ongoing conversations and discussions, such as with data entries related to the lacking biodiversity of the parking lot or the often-debated topic of domestic cats that could harm local biodiversity. These affective experiences show how closely connected these local community members are to their local land, how diverse their experiences and opinions are, and how important it is for people to see their understandings of – and relations to – biodiversity reflected in local data. Computational aesthetics, in this context, comprises much more than visual design and must carefully attend to the far more complex and dynamic experiences of local communities.

Scholarship on the aesthetics of virtual worlds have long acknowledged the wider cultural dimensions of interactive environments and the dynamic embodied interactions of people within digital spaces, which can starkly differ from more passive media such as text or film (Gigliotti 1995; Niedenthal 2009; Laurel 2013; Robson and Tavinor 2018). The sense of inhabiting and moving around in virtual environments, ranging from immersive video game worlds to simply browsing the web or scanning a QR code, involves questions of agency and subjectivity that structure user experiences and meaning making (Chang 2019; Nguyen 2020; Vella 2021). Rather than designing for aesthetic experiences solely based on technological capacities, techno-futurism, and quantitative scientific outputs, attending to the different ways in which local participants are building relationships with local biodiversity through digital data and technologies can inspire far more intimate and meaningful interactive connections to caring for local lands.

This design orientation helps to deliberately employ community data practices to further challenge the sleek futuristic aesthetic patterns that are prevalent in technoscientific innovation, fostering methods in which digital representations of ecosystems can more accurately reflect local aesthetics. However, if adopted uncritically, such participatory techniques also risk painting an overly idyllic picture of how local biodiversity is represented within digital simulations. Indeed, when asking local community members to describe biodiversity in the first phase of this project as detailed above, they used largely positive characteristics and emotional connections. Such reported perceptions of biodiversity must be complemented with other types of tensions that were not directly articulated by this community but nonetheless emerged through their engagements with biodiversity data.

Difficult situations such as being displaced, experiencing sadness over biodiversity degradation, engaging in local conflicts that directly impact the lives of community members, and dealing with negative external factors that contribute to biodiversity loss all constitute the aesthetic dimensions of interacting with local biodiversity data and were a part of this community’s reflections and conversations surrounding the data portal. Digital simulations should not just strive for technological sophistication or awe-inducing visual aesthetics but, in order to align with local experiences and encourage kinship relations with digital data, they must incorporate contextual narratives, significances, complex relations, and the full spectrum of aesthetical dimensions associated with the biodiversity data that is represented. This project shows that this can be done by making design decisions inspired by actual experiences with biodiversity and using these to challenge the more simplistic dominant technoscientific paradigms. By doing so, simulations can become more like relational living archives and reflect the ongoing environmental challenges that are affecting the stakeholders who interact with – or are impacted by – these technologies.

## Discussion: Design Recommendations for Digital Biodiversity Systems

This paper draws on ethnographic insights from an extensive participatory research project with an ecovillage community living in the Netherlands to investigate how digital biodiversity data is used and gains meaning in local restoration projects, how these experiences contrast with large-scale innovation patterns, and what new design recommendations emerge from these insights. This is important because the design and development of new global biodiversity monitoring and simulation technologies is fast-paced and tends to prioritize technological innovation and quantitative data collection. They thereby risk overshadowing digital environments that better engage with the nuanced complex relations that restoration communities have with biodiversity data.

Aligning with ongoing CSCW research and theoretical perspectives towards relational, situated, and practice-oriented research in cooperative settings to understand the socio-technical dimensions of ecological communities, this work contributes research that uncovers emerging biodiversity data practices and brings these directly in conversation with ongoing technological innovations. The design research methods employed in this project focus on understanding biodiversity monitoring practices together with the ecovillage community over a period of six months. Specifically, the use of creative practice, workshops, and open data systems propose new ways of making large-scale computational infrastructures that are still under-development experientially available and locally meaningful to enable further discussion and inform their designs. This project further articulates a growing ecological view of CSCW by employing tactics of participatory infrastructuring through activities in which communities actively engage in defining futures by creating networks, (dis)agreements, and entanglements that firstly grow socio-material practices, from which computational tools might follow (Bødker et al. 2017; Light et al. 2024).

More specifically, this work offers new empirical community-driven insights narrating what biodiversity data is regarded as relevant, at what level of detail, and in relation to which environmental phenomenon (Nadim 2021). Aligning with these theoretical perspectives, such localist views are relevant to illustrate in more detail how restoration communities work with digital biodiversity data, where new designs can emerge from restoration practices rather than remain inspired primarily by technological capacities (Westerlaken 2024). The data portal developed in this study illustrates that local data practices can inspire designs that enable flexible connections to biodiversity data and prevent users from becoming locked into fixed modes of representation and simulation. To further examine these connections, observations were analysed through the lens of digital kinships to identify meaningful human-ecosystem relations while remaining in conversation with emerging technical features. This work expands the field of CSCW by applying empirical methods and new creative practices to rapidly changing technological formations as they are being funded and developed. Moreover, it empirically illustrates how technological innovation and community-insights can be put directly in conversation with each other through participatory research and creative practice. Furthermore, this research expands existing work on participatory sensemaking with environmental technologies by further detailing examples of the socio-political issues that local communities experience in reflecting on environmental data (Niederer and Priester 2016; Dema et al. 2017). In addition to advancing research, these activities inform key design recommendations for emerging biodiversity monitoring technologies and infrastructures. Aligned with the thematic sections of the results, these recommendations are:

Designing for Environmental Storytelling:

- Digital biodiversity representations should not merely document species observations but facilitate meaningful connections between species identification and broader ecosystem health markers. This research highlights the value of connecting species data with ecosystem implications such as indicator species, pollution markers, or soil quality metrics. By making these ecological interdependencies explicit, technologies can better support community-driven restoration projects in contextualizing biodiversity data and shaping environmental narratives.
- Increasing the volume of biodiversity data does not necessarily enhance coherence in ecosystem understanding. Instead of overwhelming users with data accumulation, designs should prioritize mechanisms that allow users to selectively foreground relevant information through transparent filtering and correlation processes. This supports more actionable, localized insights rather than an excess of unstructured data.

Designing for Prediction and Future-Making:

- Biodiversity technologies should be designed not as predictive tools that dictate outcomes but as interfaces that prompt further inquiry. Rather than positioning data points as definitive conclusions, this research suggests designing ways in which environmental data can become a catalyst for expert discussion, reflection, and community-based decision-making.
- The community involved in this research showed that this can be done by designing technological features that support users to engage in"what if"inquiries rather than adhere to deterministic predictions. This includes systems that can bring data more directly in conversation with ongoing tensions, integrating discussion-oriented cues, and explicitly surfacing environmental challenges.

Designing for Dynamic Response:

- Biodiversity technologies must broaden their conceptions of interactivity to acknowledge how digital systems actively co-construct human–environment interactions. This research demonstrates that technologies shape ecological attention, determining what users notice, how they interpret environmental change, and which interventions they prioritize. These technological influences should become more transparent to users, creating critical awareness of their role in shaping restoration practices.
- Animals who interrupt monitoring and less visible species who become more noticeable through technologies demonstrated their active role in shaping restoration practices. Foregrounding these influences more as features of technologies help to reimagine digital systems as part of ecosystems rather than falsely portray such systems as separate representations or twins.

Designing for Simulation Aesthetics:

- Adapting biodiversity engagement to trends in minimalist digital aesthetics reinforces top-down and technology-centric assumptions. This research suggests the opposite approach by informing design based on the aesthetics encountered in biodiversity restoration projects so that users find their affective experiences reflected in their biodiversity data, thereby creating more resonant digital experiences.
- Instead of futuristic visuals or environmental realism there are more relevant aesthetics to incorporate in design innovation. For example, this project showed how biodiversity is less stylized/controlled and involves experiences that emerge from dealing with droughts and floodings, perceiving small every-day encounters with other species, observing things grow and decay, and experiencing loss and sadness. Technologies should more appropriately be designed for the full range of aesthetic dimensions in relation to biodiversity.

## Concluding Remarks

The insights from the ecovillage community in collecting and engaging with biodiversity data through digital technologies and creative praxis help to rethink how digital biodiversity technologies can more effectively resonate with biodiversity preservation and restoration efforts on a local level. These experiences are limited to the community in the Netherlands that participated and thus centre this community’s connections to their local biodiversity data, but they nonetheless provide actionable insights that extend beyond this project and can inform alternative digital systems as articulated in the eight design recommendations above. During this project, the community engaged with a large variety of different biodiversity monitoring techniques that created broader insights into their use of data for restoration practices, making it a suitable sample for wider user research into emerging biodiversity technologies. However, their attention to biodiversity is largely shaped through sustainability principles, technology use, and ecological landscapes that are distinctive to the geographical area where many of these new infrastructures are being developed. Undertaking this type of research with communities engaging in restoration of very different ecosystems, geographical locations, and approaches to sustainability will undoubtedly reveal additional insights that can further shape new technologies.

The rapid pace at which new biodiversity technologies are funded and developed necessitates more work that enable communities to critically and meaningfully respond to environmental technologies that are still in development but are expected to impact ecosystem monitoring. In this time of urgent environmental degradation, to remain relevant at producing critical knowledge for alternative computational systems, participatory methods will have to adapt to immediate climate issues as well as engage with complex fast-paced computational innovation. The combination of creative practices and participatory methods are specifically well-suited to connect communities to complex technological innovations because they can offer the tailor-made experiences and in-depth work through which communities can engage with the effects of emerging innovation. These processes can inspire the design of digital environments that better align with the local contexts in which they are used and emphasize the value of more community-driven models for technological innovation.

## Supplementary Information

Below is the link to the electronic supplementary material.

Supplementary file1 (MP4 271872 KB)

## Data Availability

No datasets were generated or analysed during the current study.
